# Evaluating the Global Distribution and Characteristics of Research Studies Focusing on Swine Farm Biosecurity: A Scoping Review

**DOI:** 10.1155/2024/6497633

**Published:** 2024-11-20

**Authors:** Isha Agrawal, Erin E. Kerby, Csaba Varga

**Affiliations:** ^1^Department of Pathobiology, College of Veterinary Medicine, University of Illinois Urbana–Champaign, Urbana 61801, Illinois, USA; ^2^University Library, College of Veterinary Medicine, University of Illinois Urbana–Champaign, Urbana 61801, Illinois, USA; ^3^Carl R. Woese Institute for Genomic Biology, University of Illinois Urbana–Champaign, Urbana 61801, Illinois, USA

## Abstract

Despite significant advances in swine biosecurity (BS) over the last decade, BS plans have yet to be broadly adopted on swine farms. The Preferred Reporting Items for Systematic Reviews and Meta-Analyses extension for Scoping Reviews (PRISMA-ScRs) framework was followed to review the literature, describe the worldwide distribution of publications on swine farm BS, and characterize the research methodologies used. The final data extraction and analysis included 157 publications originating from 48 countries. Several publications (*n*=93) used face-to-face interviews for data collection. An increase in the adoption of online and multimode approaches was detected after 2009. Many publications (*n*=92) focussed on the impact of BS on the incidence of swine diseases such as porcine reproductive and respiratory syndrome (PRRS) and African swine fever (ASF). Only 16 studies reported proposing incentives for study participation. Regions with high publication numbers were detected in Western and Southern Europe, Northeast of South America, and East Africa. Areas with low publication numbers were in Eastern Europe, North and Central Africa, Central America, and the Northwest of South America. This study identified the most common study methodologies used to assess swine farm BS. Countries with limited swine BS research studies were identified where future investigations are needed.

## 1. Introduction

Pork and pork products are produced and consumed worldwide, with the highest production and consumption concentrated in Asia, Europe, and North America [1]. Ranked as the most consumed meat in the world, the global demand for pork products has increased over the last decades [2]. This increased market demand created a shift in pig production systems, driving structural change from several small-scale, independent, and family-owned farms to a few large, vertically integrated, and corporate-owned swine production systems [2]. The increase in the production of pork products to meet the rising market demands and the increase in trade between countries resulted in the globalization and diversification of the swine industry [3]. However, this growth in international trade and transboundary animal movements has been implicated in the emergence and spread of infectious diseases worldwide [4]. Swine infectious diseases like African swine fever (ASF), classical swine fever (CSF), porcine reproductive and respiratory syndrome (PRRS), and porcine epidemic diarrhea (PED) are threatening the swine industry worldwide due to their health and economic burden [3].

The spread of infectious swine diseases into nonexposed herds impacts the swine industry by increasing disease mortality, morbidity, and management costs [4]. Cases like the introduction of the PED virus into the US swine population from China in 2013 through exported pork [5] and the introduction of the PRRS virus into the United States via imported infected wild boar from Europe [6] show the consequences of the disease introduction into a nonexposed swine herd and its negative impact on the health and productivity of the US swine population. The recent spread of ASF from China to 15 other Asian countries through trade, unsafe animal husbandry practices, and unregulated movement [7] is another such case. Also, the epidemiological assessment of the 2021 ASF outbreak in the Dominican Republic identified noncompliance to the recommended biosecurity (BS) practices by swine farms, increasing the introduction and spread of ASF among swine farms [8].

Globalization adds value to the agricultural economy. However, it also increases the risk of foreign animal disease introduction and spread through frequent pork and pork products–related trade [4]. This warrants the implementation of control measures both at the national and international levels to prevent infectious disease introductions into local swine populations [9]. Previous studies described the importance and effectiveness of BS practices in preventing disease introduction and spread by controlling various disease transmission routes [10]. The adoption and implementation of BS practices by swine farmers depend on individual, environmental, and legal factors. The individual factors are governed by the knowledge and attitude of the swine producers towards the risk of infectious diseases and the feasibility and perceived relevance of the BS practices in safeguarding their swine production [11, 12]. The environmental factors are mostly related to the prevalence of swine disease in the region, the availability of resources, and the climatic conditions contributing to the maintenance and spread of diseases and the adoption of BS practices [10]. The legal factors include the state and/or federal government involvement in regulating animal husbandry practices, disease reporting, and indemnity for maintaining effective farm BS practices [13, 14].

Despite variability in the swine production system worldwide, adopting effective BS practices would benefit all swine farms and productions equally [15]. The swine industry has had an ongoing conversation around BS practices for the last few decades. Researchers have assessed on-farm BS practices for different swine productions and farm types and attempted to identify challenges in encouraging farmers to adopt BS [12]. However, no single study comprehensively describes the literature on the type of BS assessment conducted worldwide, the methodology used for evaluating BS practices, and the major gaps identified during the BS knowledge, attitude, and/or practice assessments.

Publications are often considered proxies for the research priorities of academia and the scientific community. Moreover, funding agencies (government and corporate) play a critical role in shaping these research priorities by allocating funds and resources to research that addresses the industry's needs. This study utilizes a scoping review methodology to understand the current literature on BS practices on swine farms and related attitudes and knowledge of swine producers. The study focuses on the geographic distribution of the publications and the research methodology employed when assessing BS knowledge, attitudes, and practices among swine producers, veterinarians, academia, and other swine industry stakeholders.

## 2. Materials and Methods

This scoping literature review was conducted and reported following the Preferred Reporting Items for Systematic Reviews and Meta-Analyses extension for Scoping Review (PRISMA-ScR) checklist [16].

### 2.1. Data Collection

For this review, five databases, CAB Abstracts, Web of Science, PubMed, Science Direct, and Scopus, were searched for relevant research studies until May 2022. Aligning with the study objectives, relevant keywords related to “swine BS” were identified and used to search these databases. The search term keywords were combined using Boolean operators to obtain maximum publication results. The list of search terms used for each database is enlisted in Table S1. For this review, the search was limited to peer-reviewed research articles and short communications with full-text availability in English. However, no limit was imposed on the publication date and geographical location for a more comprehensive literature search [17]. The citations returned in all searches were downloaded as bibliographic text files, imported into Zotero, and categorized into database-specific folders. Later, these files were exported as comma-delimited (.csv) files, and further data processing, including screening and data extraction, was conducted using MS Excel.

### 2.2. Data Processing

#### 2.2.1. De-Duplication

All the files from all the databases underwent a de-duplication process twice, once within all the search results from each database and second within the de-duplicated pooled data set from all the database searches. The subsequent dataset was used for further screening.

#### 2.2.2. Eligibility Screening

The eligibility screening was conducted in three phases based on a set of eligibility criteria prewritten by the reviewer and evaluated, edited, and approved by two other authors. The three screening phases were title screening, abstract screening, and full-text screening. A single reviewer conducted the first two phases of the screening, and two reviewers conducted the final phase. The disputed articles during all the study phases were resolved through discussion between the two reviewers involved in the screening process.

A total of 2036 publications were subjected to title screening. The publications included in the title screening step need to meet the following criteria: must be in English and the title must indicate any one of the following study themes: BS or disease prevention and control-related studies conducted on swine farms that involved swine producers, stakeholders, and/or veterinarians, disease-specific BS practices, assessment of BS perception, knowledge, and practices, adoption of swine BS plans, survey or cross-sectional studies related to swine. The publications that were not related to swine were excluded from the study. Any publication duplicates identified during title screening were also excluded. Publications that could not be categorized based on these specified screening criteria were included in the abstract screening phase.

A total of 822 publications were included in the abstract screening step. The inclusion criteria for abstract screening were: (1) The population studied must be related to the swine industry (swine producers, stakeholders, veterinarians, or other key players), (2) indication of evaluation of swine on-farm BS, hygiene, or disease prevention practices, (3) study design limited to descriptive and analytical cross-sectional studies utilizing any survey methodology (mail, online, interviews, telephone, focus groups, and on-farm evaluation), and (4) studies collecting and evaluating on-farm BS practices or risk factors specific to a particular swine disease. The publication was included for full-text review if the abstract satisfied three of the four criteria and the eligibility criteria for the title screening. The publications evaluating risk factors associated with swine disease were included, as these factors were closely related to the lack of BS practices on the farm. Moreover, publications evaluating the knowledge and perceptions of farmers, veterinarians, or industry stakeholders on swine diseases or BS were also included. These studies were considered relevant as the perception of these critical players is known to affect the adoption and implementation of BS practices. The publications for which the abstract was unavailable or whose eligibility could not be determined during abstract screening were evaluated during the full-text screening phase.

A total of 289 publications were included in the full-text screening. Studies that did not meet the inclusion criteria specified for abstract screening were eliminated during this stage. Any study for which the full text could not be accessed through open or institutional access was excluded from the review. After the full-text review, 157 articles satisfied all the inclusion criteria for the scoping review and were included for final data extraction.

#### 2.2.3. Data Extraction

**Figure 1:**
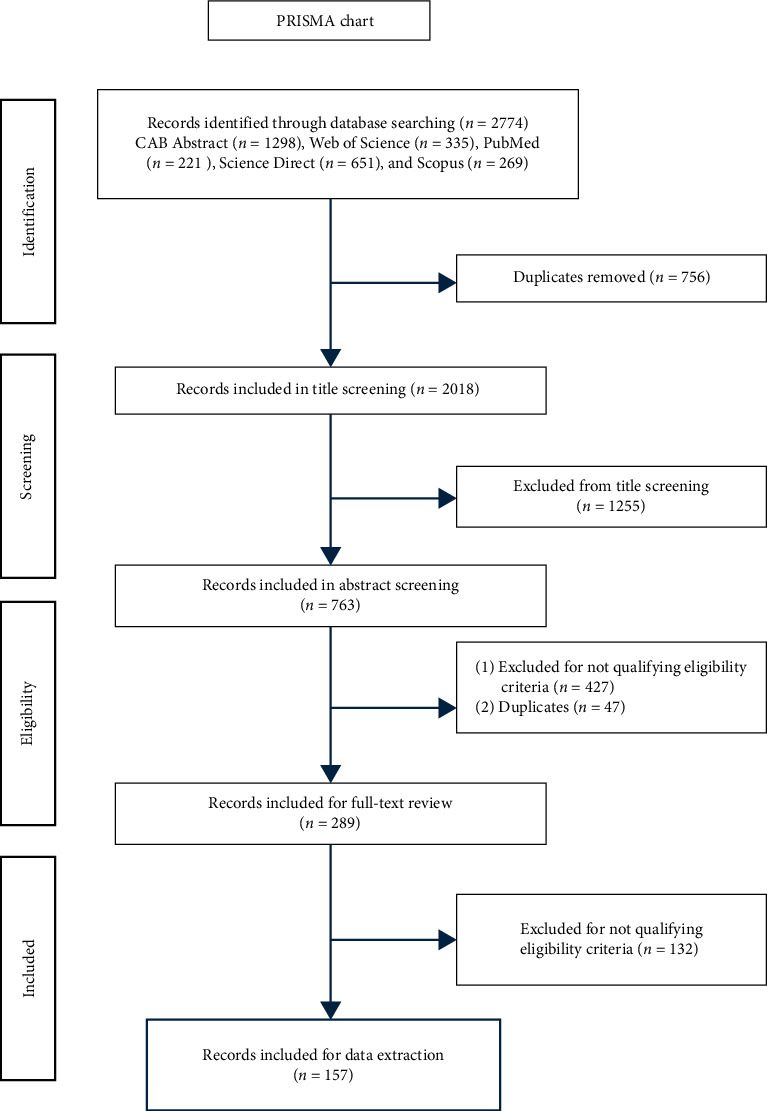
The schematic presentation of the review procedure following PRISMA-ScR guidelines. PRISMA-ScR, Preferred Reporting Items for Systematic Reviews and Meta-Analyses extension for Scoping Review.

During the study design process, a data extraction form was developed and approved by all the authors. Information on the study region (the country of the study region was recorded), country of the author's affiliation, target population, study design, survey methodology, and data collection mode was collected during the full-text screening phase. All the authors developed the above-described categories a priori following the PRISMA-ScR guidelines [16]. The study protocol is described in Figure 1.

### 2.3. Data Analysis

The data was recorded using Microsoft Excel (version 16.70, Microsoft Corporation, Redmond, Washington, USA). For statistical analysis, only descriptive statistics were computed for the extracted data. The data visualization and statistical analysis were conducted in RStudio using the “tidyverse” R-package [18].

The world shape file was obtained from a publicly available Environmental Systems Research Institute, Inc. (ESRI) database for spatial analysis. Before spatial analysis, the shape file was projected to World Fuller using ArcGIS Pro 10.7.1 (Environmental Systems Research Institute, Inc., Redlands, CA, USA). The data on the world swine population was sourced from the Food and Agriculture Organization of the United Nations (2023) [19].

A two-step spatial analysis was employed: First, an exploratory spatial analysis using choropleth maps visualized the distribution of publications globally. A fixed distance band was used for conceptualization, and a modified natural breaks (Jenks) classification method was used to isolate regions with no publications and minimize within-class and maximize between-class variance [20].

Second, a spatial statistical analysis using global and local cluster analysis. A 25 incremental distance band was used for incremental spatial autocorrelation (Moran's I method) [21] to identify distance bands with the highest global clustering to assess the global clustering of the publications. The local cluster analysis used the distance band with the highest *z*-score from the global cluster analysis [22]. Local spatial autocorrelation analysis was performed using Moran's I method [23] to determine whether the number of publications at the country level exhibited random distribution or clustered patterns [24].

ArcGIS Pro 10.7.1 (Environmental Systems Research Institute, Inc., Redlands, CA, USA) was utilized for mapping and spatial analysis.

## 3. Results

### 3.1. Publication Distribution and Trends

**Figure 2:**
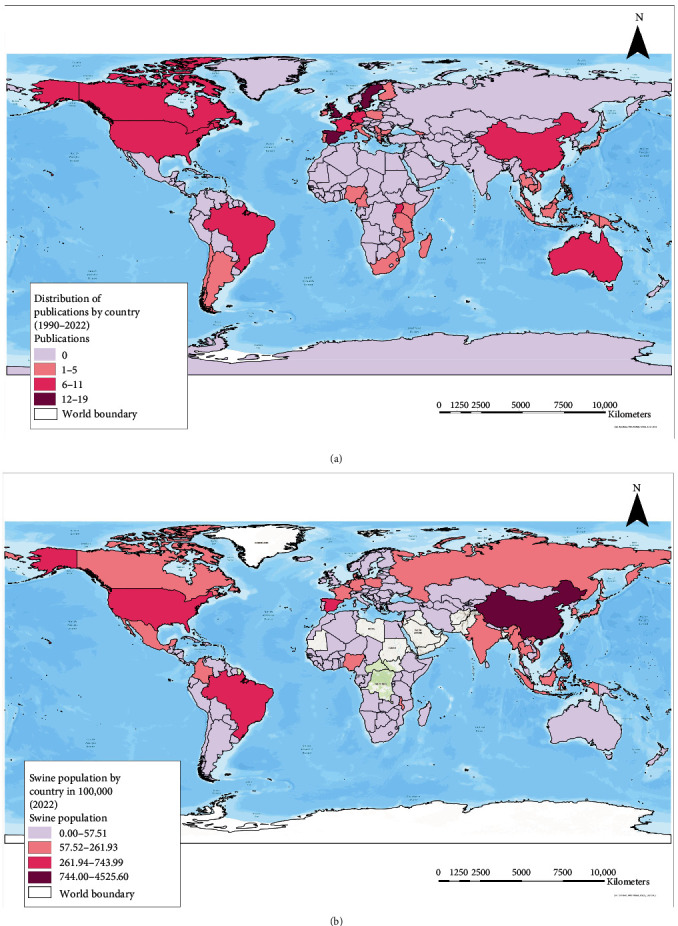
The global distribution of the (a) publications on swine BS and (b) swine population. BS, biosecurity.

The data was extracted from 157 publications from 48 countries published between 1990 and 2022.  Figure 2 shows the distribution of the publications (Figure 2a) and the background of the swine population around the globe (Figure 2b). A high proportion of studies were concentrated in European countries (notably Western and Southern Europe). Publications from the leading pork-producing countries like China (*n*=7), the United States of America (*n*=10), and Brazil (*n*=6) were moderate.

**Figure 3:**
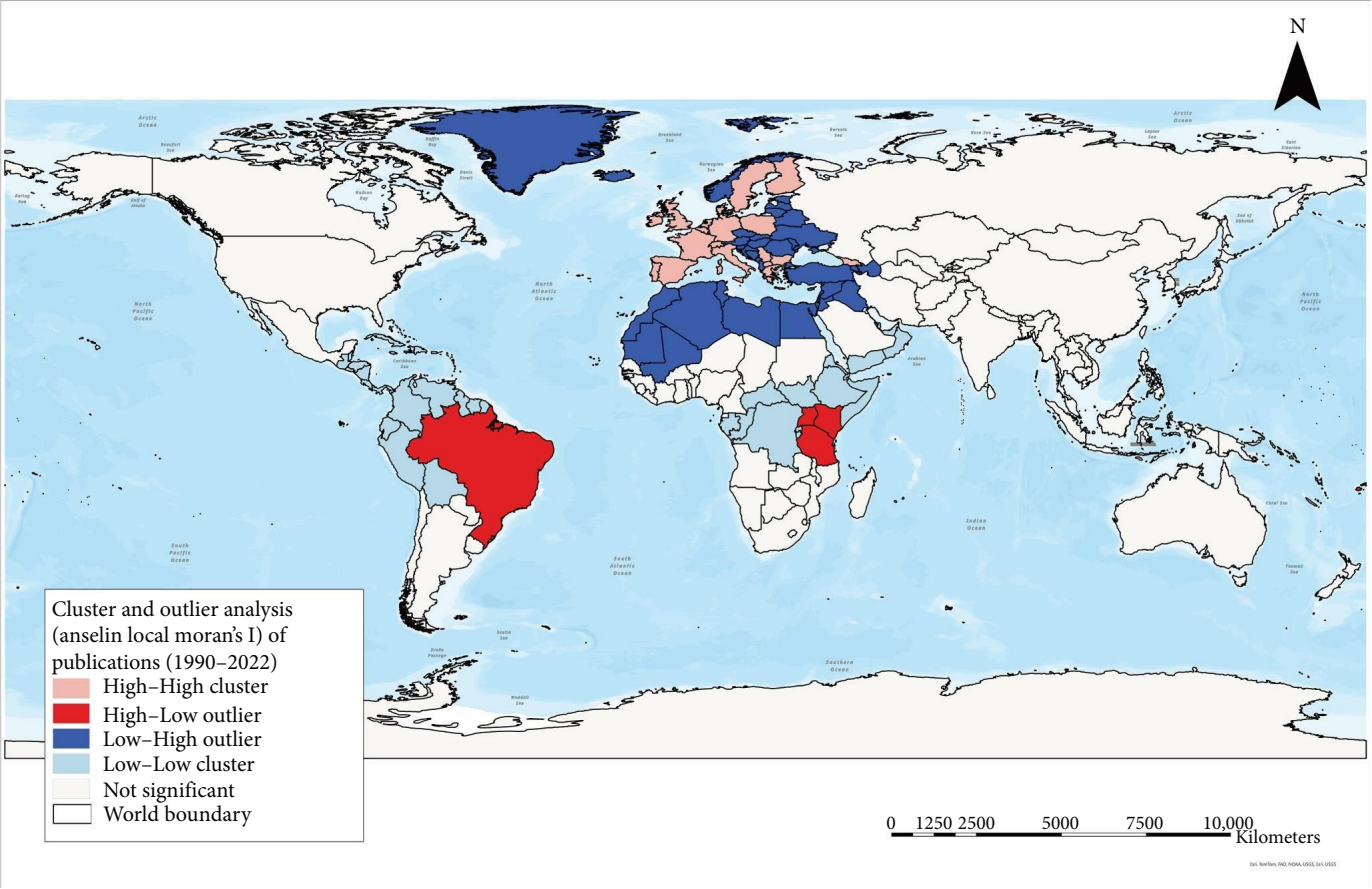
Local spatial clusters of publications related to swine BS identified by Moran's I statistics between 1990 and mid-2022. Euclidean distance band of 4165.69 km was used. Not all countries in the identified clusters, particularly the low–low and low–high clusters, could be visualized on the map, as these are mostly small island nations. BS, biosecurity.

Figure 3 describes the country-level local clusters of publication numbers across the world.

The Local Moran's I method revealed several clusters of publications worldwide. Nineteen high–high publication number clusters were identified in Europe (Belgium, Bulgaria, Denmark, Finland, France, Georgia, Germany, Greece, Ireland, Italy, Netherlands, North Macedonia, Poland, Portugal, Serbia, Spain, Sweden, Switzerland, and the United Kingdom). Five high-low publication number clusters were identified (one in North America, one in Oceania, and three in Africa), depicting regions with high publications surrounded by regions with low publications.

A total of 63 countries were included in the low–low publication number clusters, implying regions with a low number of publications were surrounded by regions with a low number of publications. Of these 63 low–low clusters, 38 clusters were reported in North America, 8 clusters in South America, 11 in Africa, and 6 in Asia (Figure 3).

Last, 52 countries were in low–high clusters, with 1 cluster in North America, 7 in Africa, 10 in Asia, and 34 in Europe. The low–high clusters suggest that regions with low publications were surrounded by regions with a high number of publications.

All identified cluster types and names from Cluster and Outlier analysis are described in Table S2.

**Figure 4:**
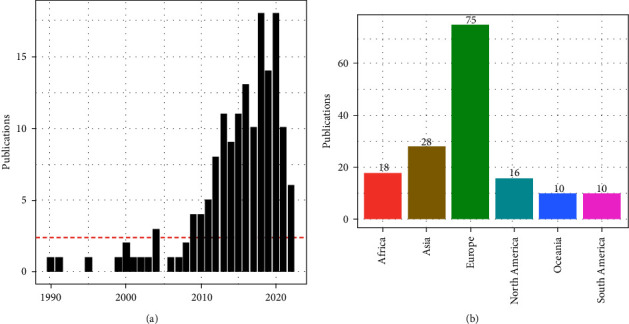
The frequency distribution of publications related to swine BS (*n* = 157) (a) by year (between 1990 and mid-2022) and (b) by continent. BS, biosecurity.

Figure 4a shows the frequency distribution of publications over the last three decades.

After 2010, an increase in publications related to swine BS was observed (Figure 4a). In the Unites States, 80% (*n*=8) of publications were observed during this period. The maximum number of publications on swine BS in the review were from Europe (*n*=75), followed by Asia (*n*=28) and North America (*n*=16; Figure 4b).

### 3.2. Study Characteristics

The study characteristics of the publication included in the review are described in Table 1.

All studies included in the review were cross-sectional studies. Four cross-sectional studies were conducted in two phases, sampling different populations in each phase.

Only 33% of the publications focused on swine BS in general, and a large segment (58.6%) focused on swine diseases, with some evaluating participants' on-farm BS practices or perceptions. Around 12% of the publications evaluated livestock, poultry, or backyard farms in combination with pig farms or as multispecies farms rearing pigs.

Of the 129 publications that mentioned their funding sources, 40 countries (*n*=12, European Union (EU) countries receiving funds from the EU) received international funding for their study. Of all the publications, 61 had one or more authors with affiliations outside the studied country. Nine of the 10 publications indicated US-based funding sources, with no funding source mentioned in one publication. All the US study publications were authored by researchers from US institutions only. However, in 10 publications, US authors collaborated on international studies worldwide.

**Figure 5:**
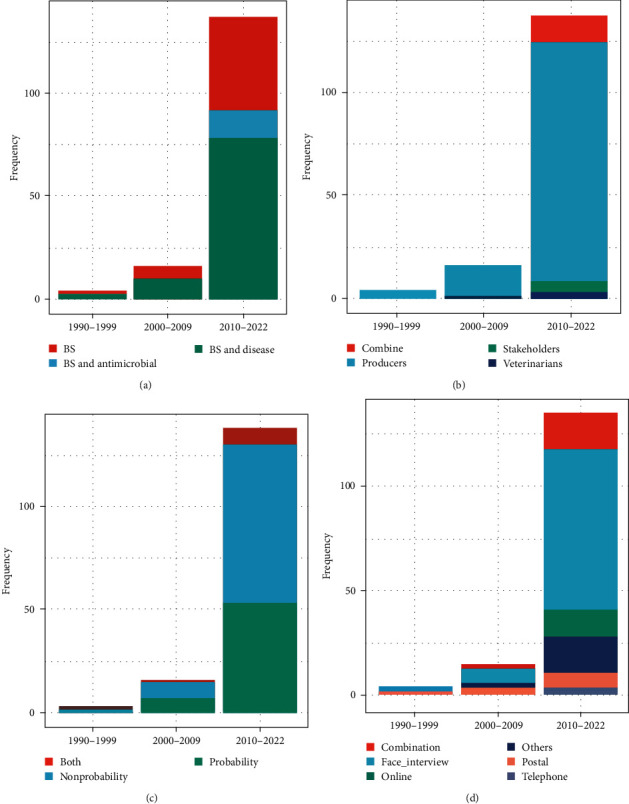
The characteristics of publications related to swine BS worldwide by decade (1990–mid-2022). (a) Study Purpose, (b) study participants, (c) study sampling strategy, and (d) data collection mode. BS, biosecurity.

Figure 5 shows the distribution of study characteristics in each decade. The publications were published between 1990 and 2022 and were categorized into three decades.

Most swine BS–related publications were conducted after 2009 (Figure 5a). A change in the publications study characteristics' was observed after 2009, with the increase in studies focusing on antimicrobial use (AMU) associations with on-farm BS practices (Figure 5a), on assessing the practices and perceptions of veterinarians and swine-industry stakeholders (Figure 5b), and on utilizing an online or multimodal approach to collect data on farm BS from the target population (Figure 5d). However, using traditional face-to-face interviews as the data collection mode remained consistent over the decades.

When recruiting study participants, the publications mainly used nonprobability or mixed sampling techniques and rarely probability sampling, with no evident change over the decades (Figure 5c).

**Figure 6:**
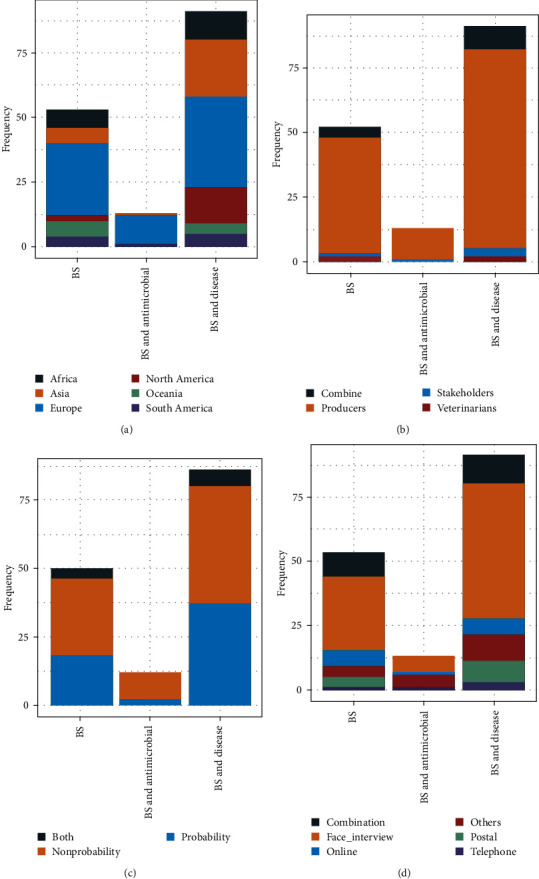
The illustration stratification of publication characteristics related to swine BS worldwide based on their study purpose: (a) the continent in which the study was conducted, (b) study participants, (c) study sampling strategy, and (d) data collection mode (others category includes focus group discussions, self or assisted paper-based or computer survey, observation-based assessment). BS, biosecurity.

Figure 6 illustrates the geographical distribution of the study characteristics of the publications categorized by the study purpose.

“BS and antimicrobial” associations were the least studied topic. This category of publications was from Europe, South America, and Asia only (Figure 6a). North America had more publications concerning “BS and disease” as the primary study purpose. The swine producers were the key focus of these three study purposes (Figure 6b). The sampling technique pattern was similar for all study purposes, except for the combination sampling approach used in “BS” and “BS and Disease” studies. (Figure 6c). Similarly, a multimodal approach was used for these two topics, with more traditional approaches in “BS and antimicrobial” studies (Figure 6d).

The face-to-face interviews (*n*=93) were conducted by either the author (*n*=13), stakeholders (*n*=4), veterinarians (*n*=11), or indicated as interviewers (either the researcher themselves or hired by the team; *n*=69). All the postal surveys (*n*=18) were accompanied by a cover letter and a prepaid return envelope.

For the online surveys (*n*=26), the link to the survey was provided through e-mail (*n*=18), instant messaging (*n*=1), in-mail (*n*=3), in-person (*n*=2), and a combination of multiple ways like newsletters, websites, journals, and social media (*n*=11). The survey web-designing tools indicated in the publications included Google Forms (*n*=2), SurveyMonkey (*n*=4), Qualtrics (*n*=2), EasyResearch (*n*=3), Questback Essentials (*n*=2), OnlineEncuesta (*n*=1), SelectSurvey.Net (*n*=1), and Thesistools (*n*=1).

Table 2 describes the characteristics of the questionnaire's structure, development, and administration used for data collection in the publications.

The questionnaires used in the publications were Biocheck.UGhent (University of Ghent, Belgium; *n*=10); BioAssest (Japan; *n*=1); Canadian Integrated Program for Antimicrobial Resistance Surveillance, CIPARS survey (*n*=1); and National Animal Health Monitoring System (US), NAHMS (*n*=1). The modified questionnaires were sourced from Biocheck.UGhent (*n*=6) and from different previously published studies (*n*=4). Two publications developed a new questionnaire for their study while utilizing an existing one for data collection. Both these publications used Biocheck.UGhent questionnaire.

The publications that pretested the questionnaire were pretested on veterinarians (*n*=14), producers (*n*=37), and stakeholders (*n*=13), alone or in combination. Seventy-three publications mentioned the questionnaire length, ranging between 6 and 610 questions. Twenty-six publications mentioned the duration of administering the questionnaire, ranging between 5 and 120 min.

Direct contact with the participants (*n*=104) was established through a listserv provided by local authorities and producers, veterinarians, and stakeholder associations and through personal contact with participants from previous projects. Moreover, 44 of the 157 publications reported assistance from veterinarians and local stakeholders in the data collection stage. Indirect administration (*n*=35) was managed through farm veterinarians, animal health workers, producers, veterinarians, and stakeholders associations' listservs and websites.

The mean number of participants surveyed in the publications was 179.85 (range: 2–1195). This value shows the number of participants who either responded to the survey or agreed to participate in the study and a cumulative of all study participants (related to swine) in publications. Fifty-three publications mentioned validating the survey responses through on-farm visual assessment (*n*=48), through telephone (*n*=2), or meeting or focus group discussions with the participant (*n*=12).

Of the 157 publications, only 16 reported offering incentives to the study participants through monetary or in-kind support. The list of incentives indicated in the publications was as follows: “MYR30 (7USD),” “AU$50 gift voucher,” “EUR330,” “$50 gift card,” “AUD$50 gift certificate and an extension information package on BS,” “AUD$50 gift certificate and printed educational material on pig farm BS,” “a lottery ticket (approximate value £ 2.50),” “£150 lottery for the first 50 replies received,” “pen and travel cost for focus group participants,” “AUD$50 gift certificate to 20 producers selected randomly from a lucky draw for five gift vouchers (each of AU $50),” “3-month free subscription to a professional pig or cattle magazine,” “a t-shirt with printed logo,” “a prize draw for £40 Amazon vouchers,” and “a lottery for a travel value cheque and a free issue of a professional journal.”

Table 3 describes the attributes evaluated by the study described in the publications, comparing global statistics with that of the United States. Twenty-seven percent of the publications evaluated participants' perceptions of BS or disease prevention measures in protecting the swine farm against diseases (Table 3).

Eighty-eight percent of the publications evaluated one or more BS-related farm practices. A publication was marked as “yes” for assessing BS score or status if they either generated a score based on the type or number of BS practices employed by the farm or if they categorized the participating farms into status based on their BS level.

Eighteen publications used the publicly available Biocheck UGhent risk-based scoring tool. In contrast, others either developed new scoring or assessment systems, such as using item response theory, experts' opinion-based multicriteria decision analysis, factor/principal/multiple component analysis, the linear scoring method, PRRS risk probability, and risk assessment scoring, or previously employed tools.

The participants' disease knowledge regarding identifying a disease symptom, its transmission, associated risk to animals and humans, or knowledge related to disease reporting was assessed. Forty-seven publications evaluated participants' disease knowledge (Table 3). Of the 157 publications, 51 reported collected samples and tested them for one or a range of pathogens for microbiological or serological examination, including PRRS virus (*n*=11), swine influenza A virus (IAV; *n*=8), CSF virus (*n*=3), pseudorabies virus (*n*=3), hepatitis E virus (*n*=2), Porcine enteric calicivirus (*n*=1), rotavirus (*n*=1), ASF virus (*n*=1), Porcine coronavirus 2 (*n*=5), Bovine viral diarrhea virus (*n*=1), *Salmonella enterica* (*n*=9), *Actinobacillus pleuropneumoniae* (*n*=4), *Mycoplasma hyopneumoniae* (*n*=3), Shiga toxin–producing *Escherichia coli* (*n*=3), *Yersinia enterocolitica* (*n*=2), *Lawsonia intracellularis* (*n*=1), *Leptospira sp*. (*n*=1), *Enterococcus sp*. (*n*=1), *Brucella sp*. (*n*=1), postweaning mortality syndrome (*n*=1), *Toxoplasma* (*n*=3), gastrointestinal parasites (*n*=3), *Trichinella* (*n*=2), *Tenia solium* (porcine cysticercosis) (*n*=2), ectoparasites (*n*=2), and endoparasites (*n*=1).

Of 10 publications on cross-sectional studies related to swine BS, nine were under the theme “BS and disease,” whereas a single publication focused solely on “BS.” In the reviewed publications from the United States, no study has explored the association between swine BS practices and antimicrobials. Eight of the 10 publications focused on the swine population, whereas two explored different livestock and poultry. Seven studies included producers as participants, whereas the remaining three included stakeholders (researchers, subject matter experts, communication practitioners, academic/extension specialists, and 4-H participants) and veterinarians.

Only one publication utilized an on-farm BS scoring tool that was a modified version of an existing scoring system. One study assessed participants' (producers, veterinarians) knowledge of a specific disease (IAV), while others (*n*=2) focused on multiple diseases. The swine pathogens investigated by publications (*n*=5) were *Brucella* sp. (*n*=1), *Salmonella* sp. (*n*=2), *E. coli* (*n*=1), and *Enterococcus sp*. (*n*=1), *Trichinella* (*n*=1), and PRRS virus (*n*=1).

## 4. Discussion

This study conducted a scoping review and assessed the current literature on the knowledge, attitudes, and practices of swine producers, veterinarians, and other stakeholders toward BS. The review examined the publications' geographical distribution, study objectives, target populations, and data collection methodologies. A large number of publications were conducted in European countries. Considering the size of swine populations, a small number of studies were conducted in leading swine-producing countries like the United States and China. The last decade has observed a gradual shift from the traditional face-to-face interview method towards a web-based or multimode survey approach.

This review identified a considerable number of publications on swine BS originating from European countries (*n*=75), spanning the period from 1990 to May 2022. Interestingly, one Eastern European country, Georgia, and the United Kingdom were identified as hot spots with a high number of studies. This heightened research interest in swine BS in these regions may be attributed to the emergence of swine pathogens such as ASF virus, CSF virus, and PRRS virus over recent decades [25–28]. To limit the impact of pathogens on swine farms, government agencies, and the swine industry provided funding to support research focused on disease prevention and control, which might explain the high number of publications on BS [26]. Conversely, a lower number of publications on BS from leading swine-producing nations like China and the Unites States were observed. This might be explained by a lack of BS research funding. Also, swine industry stakeholders might deem the existing BS measures on swine farms adequate, and research efforts are focusing more on production efficiency, disease treatment, and cost reduction rather than on assessing and improving BS protocols. However, an increase in future BS-related publications is expected from China, as this country experienced the introduction of ASF in 2018, which negatively impacted its trade and economy [7, 27, 29]. Similarly, the Unites States, as a major exporter and the second-largest producer and consumer of pork and pork products globally, faces ongoing threats from the introduction of foreign animal diseases like ASF. Additionally, the emergence of endemic swine diseases such as PRRS and PED has been observed [5, 30–32]. These disease pressures ask for increased research efforts toward swine BS. Also, the limited number of studies from African countries that are constantly fighting to control the spread of high-consequence swine pathogens [33–35] ask for increased research efforts.

A review of the methodologies adopted by researchers globally provides data-supported guidelines for future research studies. Providing incentives to obtain a higher response rate in cross-sectional surveys is believed to be effective [36]. However, of the 157 publications included in this scoping review, only 16 studies used incentives to increase study participation, suggesting a lower benefit of this approach. This finding could be explained by a lack of resources or by the researchers' belief that contacting study participants directly or via the local stakeholders and veterinarians is more effective in increasing study participation. This approach seems more personal, and using local swine stakeholder networks allows for a higher and more willful engagement in these research studies.

The majority of the publications reported using traditional face-to-face interviews to administer the survey. However, a slight deviation in this trend was observed post-2010, with an increased utilization of web-based data collection methods. Previous studies from the social sciences support this finding [37]. This could also be explained by an increase in internet usage and connectivity worldwide and the cost-effectiveness of this approach [38]. Also, online surveys allow more extensive outreach, convenient data collection and management, and efficient resource utilization [15].

In this review, the number of publications evaluating overall farm BS practices was fewer than those focussing on BS practices to control a specific disease. However, BS practices teach holistic and nonspecific protection against numerous infectious diseases by targeting their transmission pathways [10, 39, 40], and they should be implemented as the first line of defense against any type of pathogens. Also, BS measures on a swine farm require continuous evaluation and upgradation considering changes in the disease status of the region [10]. This requires a standardized tool for scoring or quantifying the current BS status of the farms. However, only a few publications reported quantifying the BS standards for swine farms using statistical analyses like factor, principle component analysis, multicriteria decision analysis, and linear scoring or using previously tested scoring systems from other publications. This highlights the need to conduct research studies that focus on developing and validating BS assessment tools while considering local disease status and swine industry structure.

A small proportion of the publications evaluated the BS-related perception or on-farm practices of veterinarians. Such publications are important as they emphasize the impact of the perception and practices of these key swine stakeholders in preventing the spread of swine diseases, as veterinarians play a crucial role in encouraging the adoption of BS practices among swine producers [10, 39–41].

This scoping review identified several knowledge gaps in the literature related to swine BS and provided methodological guidelines for future research studies. However, this review was not without limitations. First, this review included only cross-sectional studies to understand the research scope of these studies in assessing the current BS-related perception, knowledge, and practices of swine stakeholders worldwide. Any intervention or case-control study evaluating the swine farm BS was excluded from the review. Second, the review included only peer-reviewed research articles from five databases; conference proceedings, reports, gray literature, and review articles were not included. Last, only publications written in English were included, which might have excluded important articles on swine BS.

## 5. Conclusion

This scoping review evaluated the current literature on the perceptions, knowledge, and practices of swine producers, veterinarians, and other swine stakeholders regarding BS. Fewer publications were from countries leading in swine production, such as the United States and China. A shift in survey methodology from face-to-face interviews to online or mixed methodology was observed during the last decade. Incentives to increase study participation were rarely used, and researchers relied more heavily on local swine stakeholder networks to reach the study participants. The research purposes for the majority of publications were disease-centric, focusing on emerging swine diseases such as ASF and PRRS. Countries with limited research publications were identified, emphasizing critical areas for directing future research efforts.

## Figures and Tables

**Table 1:** The study characteristics of the publications included in the review (*n* = 157).

Parameters (*n* = 157)	*n* (%)
Purpose^a^	
BS study	52 (33.12)
BS and antimicrobials study	13 (8.28)
BS and disease study	92 (58.6)
Study Region	
Single nation study	138 (87.89)
Multination study	19 (12.10)
Collaboration^b^	
Yes	64 (40.76)
No	93 (59.24)
Participants^c^	
Producers	134 (84.71)
Veterinarians	4 (3.19)
Stakeholders	5 (3.18)
Combination of the above three categories	13 (8.28)
Target animal population	
Pigs	137 (87.26)
Pig and poultry	4 (2.55)
Livestock	12 (7.64)
Livestock and poultry	3 (1.91)
Backyard animals	1 (0.64)
Sampling design	
Probability	57 (36.31)
Nonprobability	81 (51.59)
Both	10 (6.37)
Did not mention	9 (5.73)
Ethics/consent requested	
Yes	85 (53.50)
No	4 (2.54)
Did not mention	68 (43.31)
Sampling frame defined	
Yes	95 (60.51)
No	45 (28.66)
Did not mention	17 (10.83)
Sample size calculation	
Yes	39 (24.85)
No	118 (75.15)

Abbreviation: BS, biosecurity.

^a^“BS” includes studies focusing solely on evaluating the perceptions and practices of study participants on swine farms related to BS or disease prevention. “BS and antimicrobial” includes studies whose primary study purpose was to evaluate antimicrobial use (AMU) and resistance (AMR) and evaluate farm BS as an alternative to AMU or as a factor in assessing AMR. “BS and diseases” includes studies that evaluate the effectiveness of disease prevention or BS practices against a specific disease agent or evaluate farm practices to identify risk factors for a disease. These studies mainly involve sampling and testing farms against single or multiple pathogens.

^b^Researchers from multiple countries working on study regions from one or multiple countries.

^c^(*n* = 156) One study did not mention any participants, as data was recorded based on observation from a single production unit. Producers include any person that indicated being in charge of farm activities and practices. Stakeholders include key players working with or within the swine industry, including subject matter experts, researchers, traders, butchers, 4-H participants, and extension workers.

**Table 2:** The descriptive summary of characteristics structure, development, and administration of the questionnaire for data collection in the publications.

Characteristics	*n* (%)
Questionnaire used for the study	
New	131 (83.44)
Existing	13 (8.28)
Modified existing	10 (6.4)
Both	2 (1.28)
Pretesting the questionnaire	
Yes	61 (38.85)
Not mentioned	96 (61.15)
Translation of questionnaire in local language (*n* = 40)^a^	
Yes	39 (97.5)
No	1 (2.5)
Number of questionnaire used (*n* = 156)	
One	143 (91.67)
Two	12 (7.69)
Three	1 (0.64)
Administering the questionnaire to the participants (*n* = 157)	
Direct contact	108 (68.78)
Indirect Contact	35 (22.29)
Both	9 (5.73)
Not mentioned	4 (2.55)
Survey response validated	
Yes	53 (33.75)
No	104 (66.24)
Reminders sent to the participants (*n* = 48)^b^	
Yes	18 (37.5)
No	30 (62.5)

^a^Translations were considered in the context of English. The remaining publications either did not require translations or did not mention translating the questionnaire into the local language (*n* = 115).

^b^Publications using postal, online, and telephone as survey modes.

**Table 3:** The descriptivestatistics of publications evaluating the on-farm perception and practices ofstudy participants related to swine BS and diseases, globally and in the United States of America.

Study characteristics	Globally (*N* = 157)	US (*N* = 10)
	*n* (%)	*n* (%)
Publications assessing BS-related perceptions of participants	43 (27.39)	5 (50)
Publications assessing BS practices of participants	139 (88.53)	8 (80)
Publications assigning BS scores or status to farms based on on-farm BS assessment	44 (28.03)	1 (10)
Publications assessing disease knowledge of participants	47 (29.93)	3 (30)
Publications assessing swine farm-disease status as reported by participants	62 (39.49)	5 (50)
Publications assessing farm-disease status through sample collection and testing	51 (32.48)	5 (50)

Abbreviation: BS, biosecurity.

## Data Availability

The data used to support the findings of this study are included in the article.

## Supporting Information

Supporting Information Table S1. Description of search terms used in different combinations during database searching. Table S2. Description of the type of clusters and country name as identified in the Cluster and Outlier Analysis (Moran's I).
