# Supplementary material for: Evaluating the Global Distribution and Characteristics of Research Studies Focusing on Swine Farm Biosecurity: A Scoping Review
Source: Transbound Emerg Dis. 2024 Nov 20;2024:6497633. doi: 10.1155/2024/6497633 (PMC12017066; doi:10.1155/2024/6497633)
Supplement: Supporting Information — Table S1. Description of search terms used in different combinations during database searching. Table S2. Description of the type of clusters and country name as identified in the Cluster and Outlier Analysis (Moran's I). [file 6497633.f1.docx]

**Supplementary materials**

**Supplementary Table 1**. Description of search terms used in different combinations during database searching

| **Databases** | **S.No** | **Search terms** | **Reason to drop/add a term** | **# of results** |
| --- | --- | --- | --- | --- |
| CAB Abstracts | 1 | **(swine* or pig*)** (Topic) and **biosecurity** (Topic) |  | 1384 |
|  | **2** | **(swine* or pig*)** (Topic) and **biosecurity** (Topic) and **survey** (Topic) |  | 262 |
|  | 3 | **(swine* or pig*)** (Topic) and **biosecurity** (Topic) and **(survey* OR questionnaire OR cross-section*)**(Topic) |  | 370 |
|  | **4** | **(swine* OR pig* )** (Topic) and **biosecurity** (Topic) and **(survey* OR Questionnaire*)** (topic) | Cross-section* was dropped because it gave irrelevant results | 351 |
|  | **5** | **(swine* or pig*) producer*** (Topic) and **biosecurity** (Abstract) and **(survey* OR questionnaire*)** (Topic) |  | 47 |
|  | 6 | **"swine producer" OR "pig farmers"** (Topic) and **biosecurity** (Abstract) and **(survey* OR questionnaire*)** (Topic) |  | 32 |
|  | 7 | **"swine producer" OR "pig farmers"** (Topic) and **(biosecurity OR prevention)** (Abstract) and **(survey* OR questionnaire*)** (Topic) |  | 56 |
|  | **8** | **veterinarian*** (Topic) and **biosecurity** (Abstract) and **(survey* OR questionnaire*)** (Topic) |  | 141 |
|  | 9 | **veterinarian* AND (pig* OR swine*)** (Topic) and **biosecurity** (Abstract) and **(survey* OR questionnaire*)** (Topic) |  | 47 |
|  | 10 | **(pig* OR swine*)** (Topic) and **biosecurity** (Abstract) and **(survey* OR questionnaire*)** (Topic) and **(veterinarian* OR profession*)** (Topic) |  | 58 |
|  | 11 | **(pig* OR swine*)** (Topic) and **(biosecurity OR prevention)** (Abstract) and **(survey* OR questionnaire*)**(Topic) and **(veterinarian* OR profession*)** (Topic) |  | 131 |
| Web of Science core collection | 1 | **(swine* or pig*)** (Topic) and **biosecurity** (Topic) |  | 971 |
|  | 2 | **(swine* or pig*)** (Topic) and **biosecurity** (Topic) and **Survey*** (Topic) |  | 138 |
|  | 3 | **(swine* or pig*)** (Topic) and **biosecurity** (Topic) and **(Survey*OR questionnaire OR cross-section*)**(Topic) |  | 60 |
|  | 4 | **(swine* or pig*)** (Topic) and **biosecurity** (Topic) and **(Survey* OR questionnaire*)** (Topic) |  | 214 |
|  | 5 | **(swine* or pig*)** (Topic) and **biosecurity** (Abstract) and **(Survey* OR questionnaire*)** (Topic) |  | 185 |
|  | 6 | **"swine producer" OR "pig farmers"** (Topic) and **biosecurity** (Abstract) and **(Survey* OR questionnaire*)** (Topic) |  | 23 |
|  | 7 | **"swine producer" OR "pig farmers"** (Topic) and **(biosecurity OR prevention)** (Abstract) and **(Survey* OR questionnaire*)** (Topic) |  | 37 |
|  | 8 | **veterinarian*** (Topic) and **biosecurity** (Abstract) and **(Survey* OR questionnaire*)** (Topic) |  | 101 |
|  | 9 | **veterinarian* AND (pig* OR swine*)** (Topic) and **biosecurity** (Abstract) and **(Survey* OR questionnaire*)** (Topic) |  | 35 |
|  | 10 | **(pig* OR swine*)** (Topic) and **biosecurity** (Abstract) and **(Survey* OR questionnaire*)** (Topic) and **(veterinarian* OR profession*)** (All Fields) |  | 54 |
|  | 11 | **(pig* OR swine*)** (Topic) and **biosecurity** (Abstract) and **(Survey* OR questionnaire*)** (Topic) and **(veterinarian* OR profession*)** (Topic) |  | 41 |
|  | 12 | **(pig* OR swine*)** (Topic) and **(biosecurity OR prevention)** (Abstract) and **(Survey* OR questionnaire*)** (Topic) and **(veterinarian* OR profession*)** (Topic) |  | 65 |
| PubMed | 1 | (swine* OR pig*) AND biosecurity |  | 840 |
|  | 2 | (swine* OR pig*) AND biosecurity AND (survey* OR questionnaire*) |  | 173 |
|  | 3 | "swine producer" OR "pig farmers" AND biosecurity AND (Survey* OR questionnaire*) |  | 23 |
|  | 4 | "swine producer" OR "pig farmers" AND **(biosecurity OR prevention)** AND (Survey* OR questionnaire*) |  | 70 |
|  | 5 | (swine* OR pig*) AND (biosecurity) AND (Survey* OR questionnaire*) AND veterinarian* |  | 26 |
| Science direct | 1 | (swine OR pig) AND biosecurity |  | 3195 |
|  | 2 | (swine OR pig) AND biosecurity AND (survey OR questionnaire) |  | 1340 |
|  | 3 | (swine OR pig) AND biosecurity AND (survey OR questionnaire) | Filters –  Article type  short communication & research articles  Subject area Agricultural and Biological Sciences &  Veterinary Science and Veterinary Medicine | 650 |
|  | 4 | ("swine producer" OR "pig farmer") AND biosecurity AND (survey OR questionnaire) |  | 195 |
|  |  | ("swine producer" OR "pig farmer") AND biosecurity AND (survey OR questionnaire) | Filters –  Article type  short communication & research articles Publication title  Preventive Veterinary Medicine, Animal, Research in Veterinary Science, | 104 |
|  | 5 | (swine OR pig) AND biosecurity AND (survey OR questionnaire) AND (veterinarian OR professional) |  | 733 |
|  | 6 | (swine OR pig) AND biosecurity AND (survey OR questionnaire) AND (veterinarian OR professional) | Filters –  Article type  short communication & research articles  Publication title  Preventive Veterinary Medicine, Research in Veterinary Science Subject areasAgricultural and Biological Sciences, Veterinary Science and Veterinary Medicine | 237 |
| Scopus |  | TITLE-ABS-KEY ( ( swine  OR  pig )  AND  biosecurity ) | Filter:  Articles | 916 |
|  |  | TITLE-ABS-KEY ( ( swine*  OR  pig* )  AND  biosecurity  AND  ( survey*  OR  questionnaire* ) ) | Filter:  Articles | 233 |
|  |  | TITLE-ABS-KEY ( ( swine*  OR  pig* )  AND  biosecurity  AND  ( survey*  OR  questionnaire*  OR  interview* ) ) | Filter:  Articles | 268 |
|  |  | TITLE-ABS-KEY ( ( "swine producer"  OR  "pig farmers" )  AND  biosecurity  AND  ( survey*  OR  questionnaire*  OR  interview* )) | Filter:  Articles | 45 |
|  |  | TITLE-ABS-KEY ( ( swine*  OR  pig* )  AND  biosecurity  AND  ( survey*  OR  questionnaire*  OR  interview* )  AND  veterinarian* ) | Filter:  Articles | 46 |
|  |  | TITLE-ABS-KEY ( ( swine*  OR  pig* )  AND  biosecurity  AND  ( survey*  OR  questionnaire*  OR  interview* )  AND  ( veterinarian*  OR  profession* ) ) | Filter:  Articles | 51 |

**Supplementary Table 2.** Description of the type of clusters and country name as identified in the Cluster and Outlier Analysis (Moran’s I).

| **Country** | **Clusters** |
| --- | --- |
| Albania | Low-High |
| Algeria | Low-High |
| Andorra | Low-High |
| Anguilla | Low-Low |
| Antigua and Barbuda | Low-Low |
| Armenia | Low-High |
| Aruba | Low-Low |
| Austria | Low-High |
| Azerbaijan | Low-High |
| Azores | Low-High |
| Bahamas | Low-Low |
| Bahrain | Low-Low |
| Barbados | Low-Low |
| Belarus | Low-High |
| Belgium | High-High |
| Belize | Low-Low |
| Bermuda | Low-Low |
| Bolivia | Low-Low |
| Bonaire | Low-Low |
| Bosnia and Herzegovina | Low-High |
| Brazil | High-Low |
| British Virgin Islands | Low-Low |
| Bulgaria | High-High |
| Burundi | Low-Low |
| Canarias | Low-High |
| Cayman Islands | Low-Low |
| Central African Republic | Low-Low |
| Colombia | Low-Low |
| Congo | Low-Low |
| Congo DRC | Low-Low |
| Costa Rica | Low-Low |
| Croatia | Low-High |
| Cuba | Low-Low |
| Curacao | Low-Low |
| Cyprus | Low-High |
| Czech Republic | Low-High |
| Denmark | High-High |
| Djibouti | Low-Low |
| Dominica | Low-Low |
| Dominican Republic | Low-Low |
| Ecuador | Low-Low |
| Egypt | Low-High |
| El Salvador | Low-Low |
| Eritrea | Low-Low |
| Estonia | Low-High |
| Ethiopia | Low-Low |
| Faroe Islands | Low-High |
| Fiji | High-Low |
| Finland | High-High |
| France | High-High |
| French Guiana | Low-Low |
| Gabon | Low-Low |
| Georgia | High-High |
| Germany | High-High |
| Gibraltar | Low-High |
| Greece | High-High |
| Greenland | Low-High |
| Grenada | Low-Low |
| Guadeloupe | Low-Low |
| Guatemala | Low-Low |
| Guernsey | Low-High |
| Guyana | Low-Low |
| Haiti | Low-Low |
| Honduras | Low-Low |
| Hungary | Low-High |
| Iceland | Low-High |
| Iraq | Low-High |
| Ireland | High-High |
| Isle of Man | Low-High |
| Israel | Low-High |
| Italy | High-High |
| Jamaica | Low-Low |
| Jersey | Low-High |
| Jordan | Low-High |
| Kenya | High-Low |
| Latvia | Low-High |
| Lebanon | Low-High |
| Libya | Low-High |
| Liechtenstein | Low-High |
| Lithuania | Low-High |
| Luxembourg | Low-High |
| Madeira | Low-High |
| Maldives | Low-Low |
| Mali | Low-High |
| Malta | Low-High |
| Martinique | Low-Low |
| Mauritania | Low-High |
| Moldova | Low-High |
| Monaco | Low-High |
| Montenegro | Low-High |
| Montserrat | Low-Low |
| Morocco | Low-High |
| Netherlands | High-High |
| Nicaragua | Low-Low |
| North Macedonia | High-High |
| Norway | Low-High |
| Oman | Low-Low |
| Palestinian Territory | Low-High |
| Panama | Low-Low |
| Peru | Low-Low |
| Poland | High-High |
| Portugal | High-High |
| Puerto Rico | Low-Low |
| Qatar | Low-Low |
| Romania | Low-High |
| Rwanda | Low-Low |
| Saba | Low-Low |
| Saint Barthelemy | Low-Low |
| Saint Eustatius | Low-Low |
| Saint Kitts and Nevis | Low-Low |
| Saint Lucia | Low-Low |
| Saint Martin | Low-Low |
| Saint Vincent and the Grenadines | Low-Low |
| San Marino | Low-High |
| Serbia | High-High |
| Sint Maarten | Low-Low |
| Slovakia | Low-High |
| Slovenia | Low-High |
| Somalia | Low-Low |
| South Sudan | Low-Low |
| Spain | High-High |
| Suriname | Low-Low |
| Svalbard | Low-High |
| Sweden | High-High |
| Switzerland | High-High |
| Syria | Low-High |
| Tanzania | High-Low |
| Trinidad and Tobago | Low-Low |
| Tunisia | Low-High |
| Turkey | Low-High |
| Turks and Caicos Islands | Low-Low |
| Uganda | High-Low |
| Ukraine | Low-High |
| United Arab Emirates | Low-Low |
| United Kingdom | High-High |
| US Virgin Islands | Low-Low |
| Vatican City | Low-High |
| Venezuela | Low-Low |
| Yemen | Low-Low |
